# Hereditary Deficiency of the Second Component of Complement: Early Diagnosis and 21-Year Follow-Up of a Family

**DOI:** 10.3390/medicina56030120

**Published:** 2020-03-10

**Authors:** Rosa Maria Dellepiane, Lucia Augusta Baselli, Marco Cazzaniga, Vassilios Lougaris, Paolo Macor, Mara Giordano, Roberta Gualtierotti, Massimo Cugno

**Affiliations:** 1 Department of Pediatrics, Fondazione IRCCS Ca’ Granda Ospedale Maggiore Policlinico, University of Milan, 20122 Milan, Italy; rosydellepiane@gmail.com (R.M.D.); lucia.base@gmail.com (L.A.B.); marco.cazzaniga1@unimi.it (M.C.); 2 Pediatrics Clinic and Institute for Molecular Medicine A. Nocivelli, Department of Clinical and Experimental Sciences, University of Brescia and Spedali Civili di Brescia, 25121 Brescia, Italy; vlougarisbs@yahoo.com; 3 Department of Life Sciences, University of Trieste, 34123 Trieste, Italy; pmacor@units.it; 4 Department of Health Sciences, Laboratory of Genetics, University of Eastern Piedmont and Interdisciplinary Research Center of Autoimmune Diseases, 28100 Novara, Italy; mara.giordano@med.uniupo.it; 5 Internal Medicine, Department of Pathophysiology and Transplantation, University of Milan, Fondazione IRCCS Ca’ Granda, Ospedale Maggiore Policlinico, 20122 Milan, Italy; roberta.gualtierotti@unimi.it

**Keywords:** complement deficiency, C2 deficiency, pneumococcal meningitis, Streptococcus pneumoniae, hypogammaglobulinemia

## Abstract

Complement deficiencies are rare and often underdiagnosed primary immunodeficiencies that may be associated with invasive bacterial diseases. Serious infections with encapsulated organisms (mainly Streptococcus pneumoniae, but also Neisseria meningitides and Haemophilus influenzae type B) are frequent in patients with a deficiency of the second component of complement (C2), but no data are available on long-term follow-up. This study aimed to evaluate the long-term clinical outcome and the importance of an early diagnosis and subsequent infection prophylaxis in C2 deficiency. Here, we report the 21-year follow-up of a whole family which was tested for complement parameters, genetic analysis and biochemical measurements, due to recurrent pneumococcal meningitis in the elder brother. The two sons were diagnosed with homozygous type 1 C2 deficiency, while their parents were heterozygous with normal complement parameters. For the two brothers, a recommended vaccination program and antibiotic prophylaxis were prescribed. During the long-term follow-up, no severe/invasive infections were observed in either patient. At the age of 16, the younger brother developed progressive hypogammaglobulinemia of all three classes, IgA, IgM and IgG. A next generation sequencing panel excluded the presence of gene defects related to primary antibody deficiencies. Our data show that early diagnosis, use of vaccinations and antibiotic prophylaxis may allow a normal life in hereditary C2 deficiency, which can be characterized using functional and genetic methods. Moreover, a periodical check of immunoglobulin serum levels could be useful to detect a possible hypogammaglobulinemia.

## 1. Introduction

The complement is a multi-functional complex system of the innate immunity comprising more than 30 proteins which are produced mainly by the liver and consist of activators and inhibitors interacting with each other to form three pathways of activation (classical, alternative and lectin) [1,2,3]. This system has an important role in host defense against infectious agents, in the removal of apoptotic cells and immune complexes, and in the modulation of the adaptive immune system [2]. Complement deficiencies are rare and often under-diagnosed disorders among primary immunodeficiencies [4,5,6,7].

Bacterial infections and autoimmune diseases are clinical conditions most frequently associated with complement defects [2]. Homozygous hereditary deficiency of each of the early proteins of the classical pathway of complement activation is strongly associated with the development of autoimmune diseases. Severe systemic lupus erythematosus (SLE) has been observed in more than 75% of all individuals with deficiency of the proteins of the first component of complement (C1) complex or with total deficiency of the fourth complement component (C4) [2,5]. In contrast, the deficiency of the second complement component (C2) is associated with a low prevalence of SLE, estimated at approximately 10% [2,5].

Patients with hereditary C2 deficiency are at risk of developing serious infections with encapsulated organisms (mainly Streptococcus pneumoniae, less frequently Neisseria meningitides and Haemophilus influenzae type B) [2,3,8] and should receive prophylactic penicillin therapy and be considered for both pneumococcal and meningococcal vaccinations [2,3,9]. Homozygous C2 deficiency has a prevalence of about 5 in every 100,000 persons in Western countries. Despite being the most frequent complement alteration, it represents less than 0.01% of the general population [3,4,10]. There are two known types of described C2 deficiency. Type 1 C2 deficiency is the most common, due to a 28-base pair deletion in the C2 gene (MIM 613927.001), and type 2 C2 deficiency is much less common, resulting from a heterogeneous group of mutations which lead to a selective block of C2 secretion [10,11,12].

Here, we report the case and the 21-year follow-up of two brothers with type 1 C2 deficiency. Patient 1 was diagnosed with complement deficiency after the second episode of pneumococcal meningitis. Patient 2, the younger brother, benefitted from familiar profiling and avoided severe infections.

## 2. Patients and Methods

### 2.1. Patients

Two Italian brothers born in 1997 and 2000 respectively from healthy, unrelated parents attended our University Hospital. Patient 1 was the first to come to our attention after the second episode of pneumococcal meningitis. Both patients were diagnosed with C2 deficiency, whereas in their parents C2 levels were near the lower limit of normal range. Forty healthy subjects (20 males and 20 females aged 4–38 years) served as normal controls and provided the ranges reported in Table 1 as normal values for the complement studies.

Upon diagnosis, both brothers were immunized with anti-Haemophilus influenzae type B conjugated vaccine (Acth-Hib^®^, Sanofi Pasteur Europe S.a.s, Lione, France), 7-13-23 valent absorbed pneumococcal polysaccharide capsular vaccines (Prevenar^®^ Pfizer S.r.l, Latina, Italy; Pneumo23^®^, Sanofi Pasteur MSD, Lione, France), quadrivalent meningococcal conjugate vaccine (Menveo^®^, MenACWY-CRM; Novartis Vaccines and Diagnostics S.r.l., Siena, Italy) and with the conjugate vaccine against N. meningitides serogroup B (Bexero^®^, Novartis Vaccines and Diagnostics S.r.l., Siena, Italy) as soon as it became available. They regularly carried out recalls for pneumococcal and quadrivalent meningococcal vaccines.

Antibiotic prophylaxis was also started, initially with intramuscular benzathine benzylpenicillin every 21 d, replaced by oral phenoxymethylpenicillin in 2002 and currently with oral amoxicillin in a single daily dose.

### 2.2. Laboratory Studies

#### 2.2.1. Hemolytic Assay for Functional Activity of the Classical Complement Pathway

Functional activity of complement was measured by means of complement hemolytic activity 50% (CH50). This assay was done as previously described [13,14]. Briefly, antibody sensitized sheep erythrocytes (EA) were prepared with a sub-agglutinating amount of rabbit IgM antibodies. Hemolytic activity was evaluated by mixing dilutions of test sera in glucose veronal-buffered saline (GVBS) with 50 μL of 1% EAC1-3b to a final volume of 250 μL. After incubation at 37 °C for 30 min, red cell lysis was calculated by measuring the OD415. Hemolytic activity was expressed as a percentage of lysis induced by water. 

#### 2.2.2. C2 Specific Activity

C2 specific activity was calculated using hemolytic assay in which the recovery of complement activity of a serum from a patient with a well characterized deficiency of a single complement component is obtained by adding the patient serum, as previously described [15].

#### 2.2.3. Functional Immuno-Enzymatic Assay for the Classical Complement Pathway

The functional test for the classical complement pathway was performed using a Wieslab Complement System kit (Euro-Diagnostica, Malmö, Sweden), following the manufacturer’s instructions. The wells of the microtiter strips were coated with a specific activator of the classical complement pathway, and the serum was diluted in a buffer containing specific blockers of the other two complement pathways in order to ensure that only the classical pathway was activated during incubation. The wells were then washed, and C5b-9 was detected with a specific alkaline phosphatase-labelled antibody against the neoantigen expressed on C9 during C5b-9 formation. After a further washing step, the specific antibodies were detected by means of incubation with the alkaline phosphatase substrate solution. As the amount of complement activation correlates with color intensity and is measured in terms of absorbance, the results are expressed as percentages of the activity of a standard sample (i.e., normal pooled serum fixed at 100%). This method allows evaluation of complement activity detected in serum through classical pathway activation using the terminal complement complex C5b-9 as detection system [16].

#### 2.2.4. Genetic Analysis

Genomic DNA was amplified using PCR with primers designed to specifically amplify exon 6 (ex6F: 5’-GCCTGGGCCGTAAAATCCAAATCCA-3’ and Ex6R: 5’-GCACAGGAAGGCCTCTGCTGCAGGC-3’), including the most common Type I C2 deficiency mutation [10,11,12] under standard condition. The PCR products were visualized on a 2% agarose gel and purified using ExoSAP-IT enzymatic PCR clean-up system (Affymetrix, Santa Clara CA). The purified products were then sequenced with a Big Dye Terminator kit (Applied Biosystems, Foster City, CA) and analyzed on an ABI PRISM 3100 Genetic Analyzer (Applied Biosystems, Foster City, CA).

## 3. Results

### 3.1. Clinical Features

Patient 1 is currently 24 years old; he was born to unrelated Italian parents at 40 weeks of gestation after an uneventful pregnancy. At 12 months of age, he suffered from pneumococcal meningitis with a delayed cerebrospinal fluid (CSF) sterilization (15th d), resulting in bilateral sensorineural hearing loss (SNHL). At 32 months of age, the patient suffered a second episode as meningo-encephalitis with isolation of S. pneumoniae from the CSF. Despite CSF sterilization after 5 d of antibiotic treatment, encephalic foci persisted causing a mild right cerebellar lesion.

Possible risk factors for recurrent bacterial meningitis were investigated, such as anatomic defects of cribriform plate or paranasal sinus and asplenia, with negative results. In-depth investigations to rule out primary immunodeficiencies showed normal values of serum immunoglobulins, IgG subclasses, T and B cell number and activity. Detailed functional activity of the classical pathway of the complement system was measured, revealing a reduced activity due to C2 deficiency. 

Patient 2, current age 21 years, had the same diagnosis of C2 deficiency from the age of 6 months after the diagnosis in his brother. He was born at 40 weeks of gestational age after a physiologic pregnancy. The patient did not experience any severe or invasive infection before diagnosis of C2 deficiency was made.

### 3.2. Laboratory Studies

As reported in Table 1, Patient 1 and Patient 2 showed a level of 0 for CH50 and a marked reduction of classical pathway activity measured with the immunoenzymatic method (3% of normal). The parents showed slightly reduced CH50 levels and normal levels of classical pathway activity measured with the immunoenzymatic method. 

Further analysis performed with a recovery test of complement activity showed C2 deficiency in the siblings with levels of 0 and C2 levels near the lower limits of normal subjects in the parents. 

**Figure 1:**
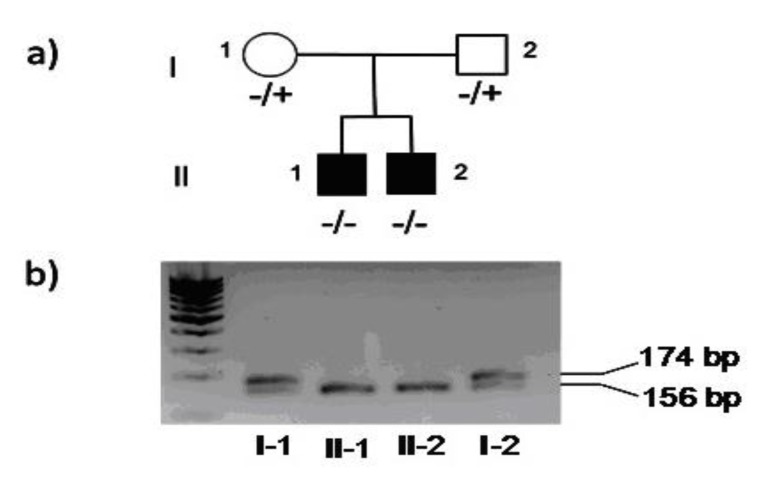
(**a**): pedigree of the family (+: wild type allele; −: mutated allele). (**b**): gel electrophoresis of PCR products, in which I-1 and I-2 (parents) showed two bands (wild type allele of 174 bp and the deleted allele of 156 bp), whereas II-1 and II-2 (children) showed only the 156 bp band.

The amplified DNA visualized on agarose gel showed two bands in the parents, representing the wild type and the deleted allele of 174 bp and 156 bp, respectively. In the two children only a band of 156 bp was present (Figure 1).

**Figure 2:**
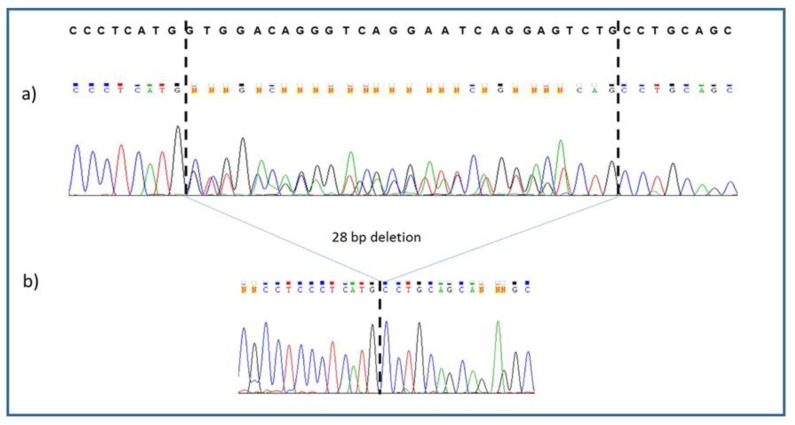
Electropherogram of the mutated allele at the (**a**) heterozygous state (parents) and (**b**) at the homozygous state (children). The reference wild-type sequence is reported in the upper part of the figure.

Sequence analyses of exon 6 of the C2 gene in all the family members revealed in the two parents the presence of the 28-base pair deletion at the heterozygous state. The two affected siblings (Figure 2) were both homozygous for the deletion.

### 3.3. Follow-Up

#### 3.3.1. Patient 1

During follow-up, Patient 1 did not suffer from severe/invasive infections. Due to recurrent episodes of rhinitis, allergological evaluation was performed at 6 years of age, which showed elevated IgE levels with specific IgE positivity for Alternaria and mites. A CT scan of the facial mass and the petrous rocks showed adenoiditis and chronic sinusitis. Although the symptoms were partially controlled by frequent nasal rinses and topic steroid cycles (based on seasonal allergen pattern), at the age of 7 years the patient underwent adenoidectomy and enlargement of the sphenoid sinus ostium. 

Due to the severe SNHL following the first episode of pneumococcal meningitis, at the age of 14 he underwent cochlear implant surgery without complications. Brain MRI has remained stable over time. The patient has always shown normal levels of IgA, IgM, IgG, IgG subclasses and lymphocyte subpopulations as well as the development of protective specific antibody titers after vaccinations (tetanus, pneumococcus, rubella, mumps, measles and hepatitis B).

#### 3.3.2. Patient 2 

Patient 2 has never experienced severe infections. Like his brother, he presented with recurrent episodes of rhinitis leading to the diagnosis of allergies to grass; nasal polyposis was diagnosed at the age of 13 years. The patient performed regular annual controls with immunological evaluation: at the age of 3 years, IgG levels were at the lower limit of normal values for his age (423 mg/dL; normal values: 462–1710 mg/dL) with normal IgG subclasses, IgA and IgM. The antibody response after a tetanus vaccination was normal. At the age of 16 years, the patient showed low immunoglobulin serum levels for all classes (IgG 363 mg/dL, normal values: 640–1909 mg/dL, IgA 38 mg/dl, normal values: 61–301 mg/dL; IgM 30 mg/dL, normal values: 59–297 mg/dL) and low IgG1 subclass (249 mg/dL; normal values: 315–855 mg/dL), with an adequate antibody response to anti-pneumococcal vaccination, normal T cell distribution and functional evaluation, as well as normal peripheral B cell maturation with switched memory B cells being at the lower limit of normal values for his age (3.6%, normal values: 3.0–46.0%). No evidence of autoimmunity was found. The patient did not show any further alteration of the immunological profile, maintaining a good health status.

A next generation sequencing (NGS) panel of known genes associated with hypogammaglobulinemia, agammaglobulinemia and common variable immunodeficiency (CVID) was performed and did not reveal the presence of any pathogenetic variant. In particular the following genes were evaluated: AICDA, BLNK, BTK, CARD11, CD19, CD79A, CD79B, CD81, CECR1, CR2, CTLA4, CTPS2, CXCR4, DKC1, DNMT3B, FCN3, GATA2, ICOS, IGHM, IGKC, IGLL1, IL21R, INO80, LRBA, LRRC8A, MAGT1, MOGS, MRE11A, MS4A1, MSH6, NBN, NFKB1, NFKB2, PIK3CD, PIK3R1, PLCG2, PMS2, PRKCD, RAG1, RAG2, SH2D1A, STAT3, TCF3, TNFRSF1 3b(TACI), TNFRSF13C, CD40, TNFSF12, CD40LG, TPP2, TRNT1, TTC37, UNG2, XIAP.

## 4. Discussion

This study, in which we report the case and the 21-year follow-up of two brothers diagnosed with homozygous type 1 C2 deficiency and of their parents, shows that early diagnosis, use of vaccinations and antibiotic prophylaxis may allow a normal life in hereditary C2 deficiency. Moreover, hypogammaglobulinemia may complicate the clinical course of the disease. Deficiency of complement components represents approximately 1–6% of all primary immunodeficiencies, but these may increase up to 10% in certain communities [12,17,18,19,20]. The frequency of congenital complement deficiency has been calculated to be about 0.03% in Western countries and Japan, excluding mannose binding lectin (MBL) deficiency, which has been estimated to occur in its recessive form in about 5% of the population [7,12,21]. Type 1 C2 deficiency is the most common complement alteration, due in 95% of cases to a homozygous 28-bp deletion in the C2 gene [10,11,12]. The here-described patients were homozygous for this mutation and the two parents were heterozygous. A less common type 2 C2 deficiency that represents 5–7% of the cases is caused by other mutations, such as 2-bp deletion in exon 2 or missense mutations [22]. In a recent study conducted in France, out of 163 children with invasive pneumococcal disease, 10% had primary immunodeficiencies and three cases had complement fraction deficiency (two C2, one C3) [23].

The mild hypogammaglobulinemia presented by Patient 2 has worsened over time, with deficiency in all the three classes of immunoglobulins (IgA-IgM-IgG) by the age of 16, without any further immunological alteration or severe infection, and thus not fulfilling the criteria of the European Society for Immunodeficiencies for CVID [24,25,26]. Consequently, the patient has not been treated with immunoglobulin replacement and has been regularly monitored for immunoglobulin serum levels. Substitutive therapy with immunoglobulins could be considered in the future if IgG levels fall further to levels that are too low to ensure effective protection against infections. In addition, antibiotic prophylaxis may have reduced the bacterial infectious incidence. In C2 deficiency, vaccinations against capsulated bacteria and antibiotic prophylaxis are extremely important in reducing serious infections [2,3,8,9]. Early diagnosis and treatment strategy in our two patients have allowed them to conduct a normal life for at least 21 years, free of serious infections and with an overall good quality of life. Particularly for Patient 2, early diagnosis was a great benefit, because the same program of vaccination and antibiotic prophylaxis of the brother was started before the presentation of invasive bacterial infections.

Association between C2 deficiency and antibody deficiency has only been reported in a very limited number of cases [27,28] and discussed recently by other authors [29,30].

It is known that the classical complement pathway has a major role in B-cell development and differentiation: C2 seems to play a direct role in the production of IgG4 and IgD and the classical pathway per se contributes to the development of a normal lymphonode germinal center, as demonstrated by the observation that knockout mice for complement C3 receptor CD21/CD35 have an impaired B-cell differentiation [29]. Moreover, only one case of congenital C2 deficiency associated with CVID has been reported in literature. That case, however, showed a high degree of consanguinity because the parents were siblings and authors did not exclude that the patient could have inherited two unlinked abnormal genes [27]. In 1978, Agnello described another patient with C2 deficiency and isolated IgG1 deficiency [28].

## 5. Conclusions

Our report supports the utility of searching for complement deficiency as part of the immunological screening in all cases of invasive bacterial disease in which favoring anatomical causes are excluded. The long-term follow-up without any infection in our two patients with hereditary C2 deficiency indicates that an early diagnosis of complement deficiency allows a prompt intervention with vaccinations and antibiotic prophylaxis, which in turn avoids infections and thus improves the quality of life. Although hypogammaglobulinemia is not a classical immunological finding in hereditary C2 deficiency, our data along with those of others suggest the need of further longitudinal studies in patients with hereditary C2 deficiency to better define this possible association.

## Figures and Tables

**Table 1:** Complement activity of the family.

	CH50 (U/mL)	Classical Pathway Activity (% of Normal)	C2 (μg/mL)
**Father**	876	100	8
**Mother**	684	84	12
**Patient 1**	0	3	0
**Patient 2**	0	3	0
Normal values *	900–1300	69–129	10–30

CH50: 50% hemolytic complement activity; classical pathway activity: immunoenzymatic method; C2: complement component 2. * 95% confidence interval of the values of the control group.
